# A Rare Case of Right-Sided Chylothorax Following Thoracoscopic Sympathectomy Due to an Anomalous Thoracic Duct

**DOI:** 10.7759/cureus.69726

**Published:** 2024-09-19

**Authors:** André Amate Neto, Amanda Tollini de Moraes, Felipe Ramos Camargo Preto, Sarah Lopes Salomão, Tales Rubens de Nadai

**Affiliations:** 1 Department of Thoracic Surgery, Bauru Medical School, University of São Paulo, Bauru, BRA; 2 Department of Medicine, Bauru Medical School, University of São Paulo, Bauru, BRA

**Keywords:** case report, chylothorax, hyperhidrosis, sympathectomy, thoracic surgery

## Abstract

Chylothorax, despite being a common complication after thoracic surgery, is rare after thoracic sympathectomy, especially on the right side of the thorax. We present a case of a patient who developed a right chylothorax after a thoracoscopic sympathectomy due to the presence of an anomalous thoracic duct located on the right side of the patient’s chest. A 37-year-old woman underwent a bilateral video-assisted thoracic sympathectomy for the treatment of primary focal axillary hyperhidrosis. During the postoperative period, there was an excessive discharge of a white, milky fluid through the chest drain, with an average daily output of 350-500 mL/day. Chylothorax was confirmed after laboratory analysis, which revealed a triglyceride level of 146 mg/dL. Due to the worsening appearance of the pleural fluid and the increased drainage volume, reaching 1,000 mL, the patient underwent exploratory videothoracoscopy. During the procedure, a lymphatic fistula was visualized in the region of the sympathetic chain, allowing the identification of an anomalous thoracic duct on the right side of the patient's thorax. The anomalous thoracic duct was dissected, with inferior and superior clipping of the duct. The patient remained stable and was discharged three days after the procedure. This case report describes an especially rare presentation, being one of the few cases of right chylothorax after thoracoscopic sympathectomy described in the literature to date. This study points out that, despite thoracic sympathectomy being considered a safe surgical procedure, unusual complications, such as chylothorax, and anatomical variations of the thoracic duct must be considered.

## Introduction

Thoracoscopic sympathectomy is a highly recommended minimally invasive surgical procedure for treating disorders related to the sympathetic nervous system, such as primary focal hyperhidrosis (PFH), a condition characterized by excessive sweating in specific areas of the body, most often the axillae, palms, soles, and craniofacial region [1,2].

Surgery is typically performed through small incisions in the chest wall, usually between the third and fourth ribs. A thoracoscope is inserted through one of the incisions to visualize the sympathetic chain along the spine, and through the other incisions, the sympathetic nerves responsible for the symptoms are resected or cauterized, with the objective of reducing sympathetic overactivity and, in the case of PFH, decreasing excessive sweating [3]. Once the procedure is complete, the thoracoscope and instruments are removed, and the small incisions are closed, typically with sutures or adhesive strips.

Although thoracic complications such as hemothorax and pneumothorax occasionally occur after thoracoscopic sympathectomy, chylothorax is a rare complication, occurring postoperatively in approximately 0.5% of all intrathoracic procedures [4]. It is especially rare on the right side of the thorax, with very few cases reported in the literature [1]. 

Chylothorax typically occurs when the thoracic duct (TD), the largest lymphatic vessel in the body, responsible for collecting lymph from smaller lymphatic vessels and draining approximately 75% of the body's lymph, is injured. When the TD is damaged, chyle leaks into the pleural cavity, leading to the accumulation of fluid, which can cause respiratory difficulties and compromise the patient's nutritional and immune status [5].

Anatomical variations of the TD are significant due to its vulnerability to injury during surgical procedures. One such variation is the right-sided TD, a rare condition in which the duct, instead of following its usual path on the left, drains into the venous system on the right. Typically, the TD ascends through the thorax, crossing from right to left at the T5-T6 vertebrae, and empties into the left venous angle. However, in some cases, it remains on the right and drains into the right venous angle, which is considered an anatomical anomaly [4,5]

In this article, we report the case of a patient who developed right-sided chylothorax following thoracoscopic sympathectomy due to the presence of an anomalous TD located on the right side of the patient’s chest. This reported case is an especially rare presentation, being one of the few cases of right-sided chylothorax after thoracoscopic sympathectomy described in the literature to date [6].

## Case presentation

A 37-year-old woman sought medical attention with a complaint of excessive axillary sweating. The patient had no underlying medical conditions and denied a history of recent illness. On physical examination, bilateral focal axillary hyperhidrosis was observed. Therefore, surgical treatment was indicated. She underwent video-assisted bilateral thoracic sympathectomy through the intercostal space between the fourth and fifth ribs, without any intraoperative complications. A chest tube was placed at the time of the operation. Postoperatively, she remained stable but complained of significant pain at the drain incision.

**Figure 1:**
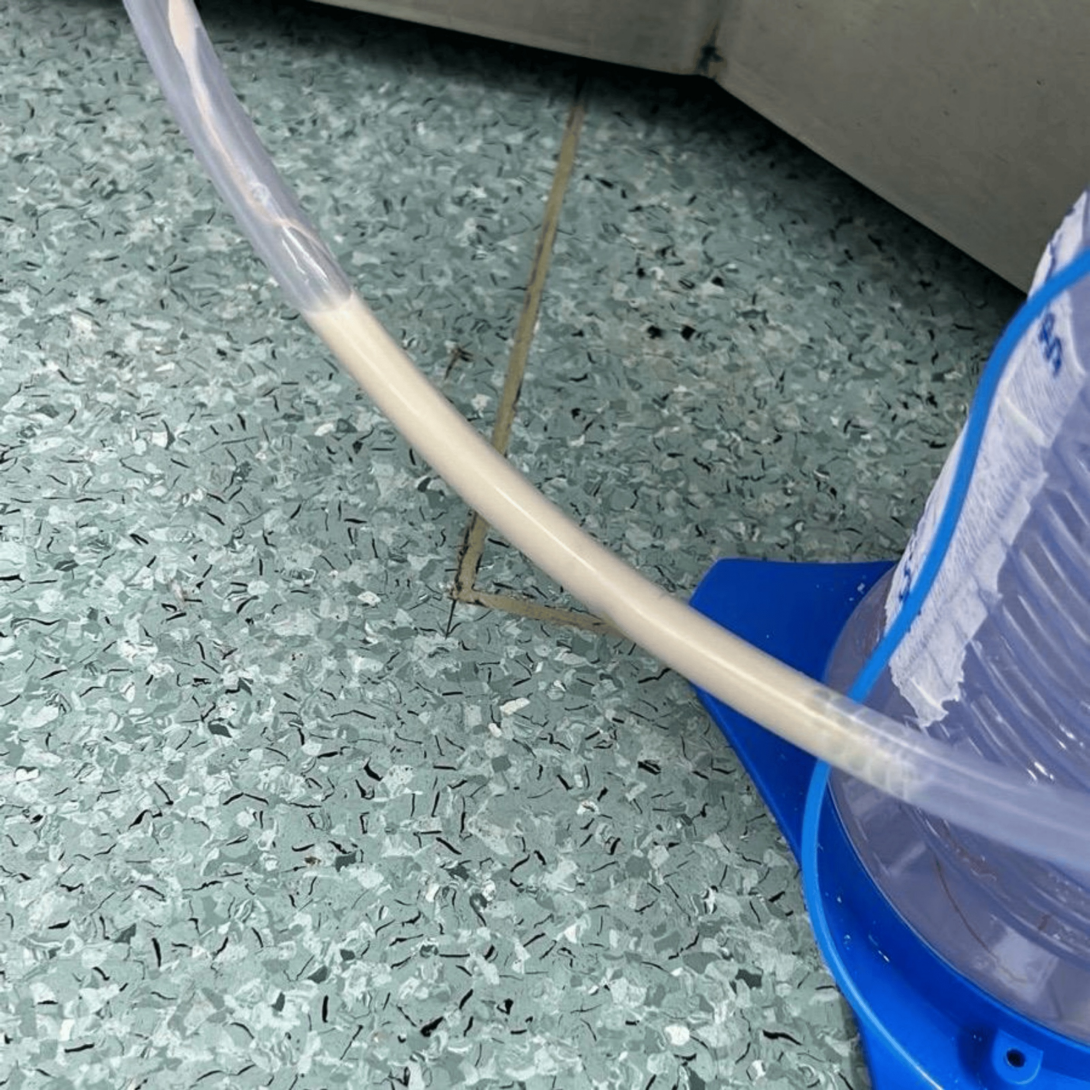
Chylous output in the chest drain Chylous fluid outflow from the patient's chest drain.

The next day, there was an excessive discharge of white, milky fluid from the chest tube (Figure 1). This finding raised suspicion of chylothorax, and the fluid was sent for laboratory analysis, which revealed a triglyceride level of 146 mg/dL, confirming the diagnosis of chylothorax, as the diagnostic cutoff is 110 mg/dL of triglycerides.

In the first seven days of follow-up, the average daily drainage was between 350 and 500 mL/day. On the eighth postoperative day, after transitioning from a low-fat diet to a general diet, the patient presented a chylous output of 1,000 mL. Due to the worsening appearance of the pleural fluid and the increase in the drainage volume, exploratory videothoracoscopy was indicated to ligate the lymphatic fistula and treat the post-sympathectomy chylothorax.

**Figure 2:**
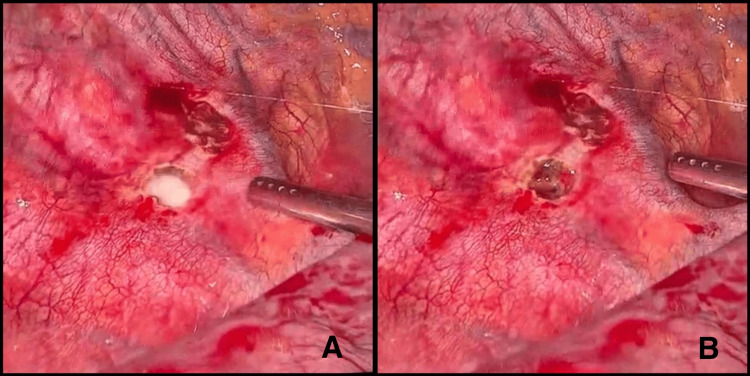
Lymphatic fistula Chyle discharge in the sympathetic chain region through a lymphatic fistula, allowing for the identification of an anomalous thoracic duct (A). Lymphatic fistula following aspiration of chylous fluid (B).

On the ninth postoperative day, the patient underwent exploratory videothoracoscopy. After the infusion of 200 mL of oil through the nasogastric tube, 90 minutes waited for the evident exit of the chyle through the chest drain. During videothoracoscopy, chyle exit was visualized in the region of the sympathetic chain, allowing the identification of an anomalous thoracic duct on the right side of the patient's thorax (Figures 2A, 2B).

**Figure 3:**
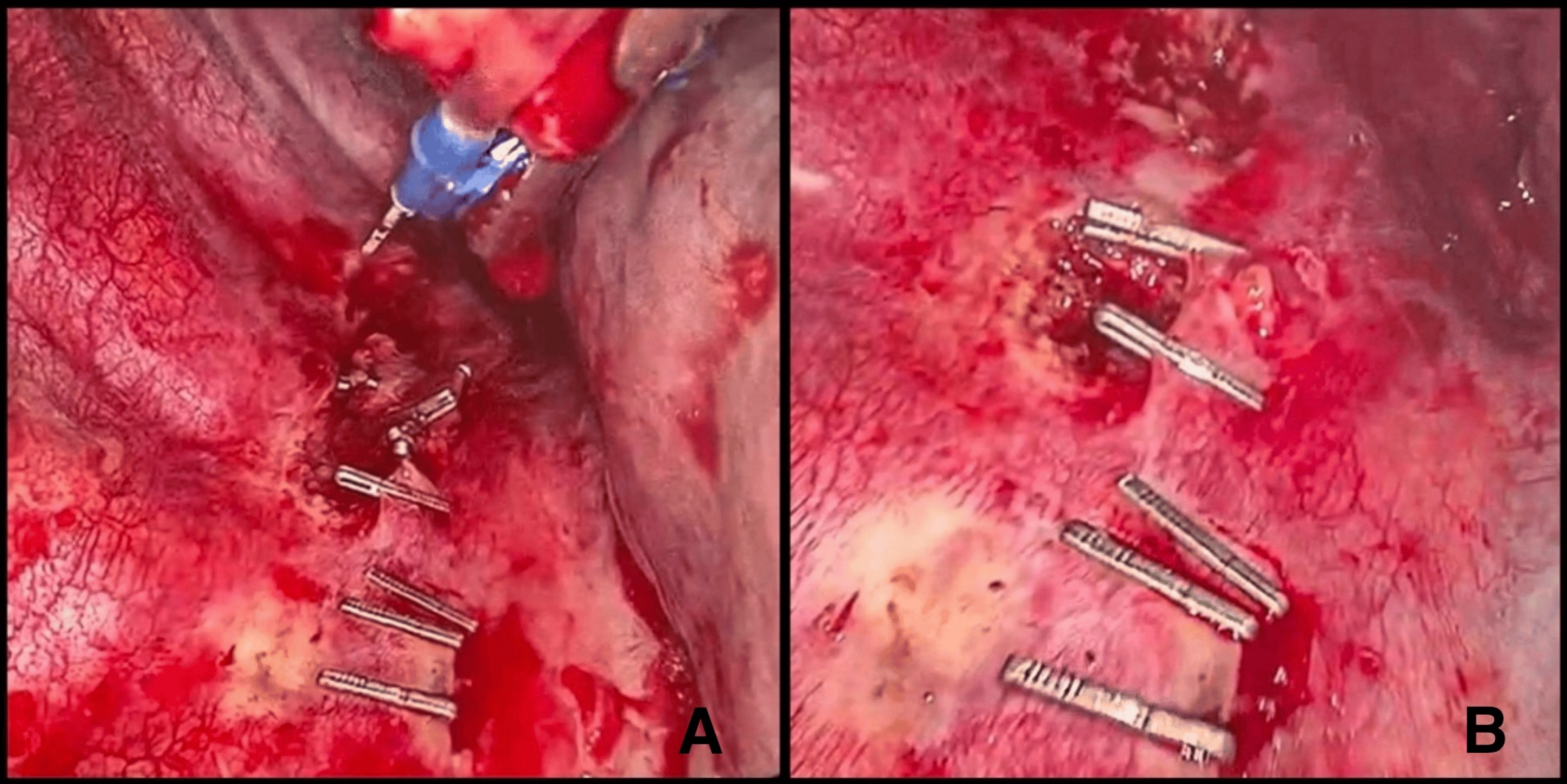
Clipping of the anomalous thoracic duct Dissection of the anomalous thoracic duct, with inferior and superior clipping (A). Result of clipping of the anomalous thoracic duct (B).

The anomalous thoracic duct was dissected, with inferior and superior clipping of the duct (Figures 3A, 3B). The surgery was completed with the insertion of a chest drain and layered closure of the wound. No complications occurred intraoperatively. During postoperative follow-up, the patient remained stable and on a low-fat diet. Daily drainage was between 50 and 75 mL/day, and the chest tube was removed on the third postoperative day. The patient was discharged without any further complications.

## Discussion

Chylothorax, although a common complication after thoracic surgery, is rare following thoracic sympathectomy, with limited reports in the literature, particularly on the right side of the thorax. In a review of the PubMed database, we identified only one case report of right-sided chylothorax after sympathectomy [1], with a similar approach to our case. Sapmaz et al. reported a case of a 23-year-old male who developed right-sided chylothorax after thoracic sympathotomy. Diagnostic video-assisted exploration of the right hemithorax revealed a chyle leak at the R4 level, which was controlled with vascular clips [1].

Hyperhidrosis is a disorder characterized by excessive and uncontrollable sweating, diffuse or localized, beyond the body's physiological needs. It can be classified as primary or secondary [2]. Primary hyperhidrosis is idiopathic and related to the hyperactivation of sympathetic nerves, typically affecting specific areas of the body, most often the axillae, palms of the hands, soles of feet, or the craniofacial region. Secondary hyperhidrosis usually results from complications of underlying diseases or medical conditions. Although not fatal, hyperhidrosis has a substantial impact on patients' lives, causing embarrassment and psychosocial disorders, which result in reduced quality of life [2,3].

Conservative treatments for PFH do not guarantee satisfactory results, with success rates considerably lower compared to the curative outcomes of surgical procedures [3]. Therefore, blocking the thoracic sympathetic branches through thoracic sympathectomy or sympathicotomy is considered the most widely accepted treatment for PFH. Sympathicotomy is regarded as the optimal treatment for primary hyperhidrosis due to its shorter operative time, reduced compensatory sweating, and fewer adverse effects related to compensatory sweating. This procedure is considered less invasive than sympathectomy, as it involves cutting part of the nerve rather than removing or destroying a portion of the sympathetic chain. These procedures are performed thoracoscopically, typically accessing the sympathetic chain between the third and fourth ribs [1,7].

Among the advantages of video-assisted thoracoscopic surgery (VATS) are a shorter hospital stay, lower intensity of postoperative pain, faster recovery, and better aesthetic results [3]. However, in addition to frequent complications such as pneumothorax and compensatory hyperhidrosis, this procedure can present atypical complications, including Horner's syndrome, large vessel injury, and brachial plexus injury [1,8]. Chylothorax is a rare complication following thoracic sympathectomy. This condition is caused by obstruction or extravasation of the TD or its branches, leading to the accumulation of lymphatic fluid in the pleural cavity [9].

The origin of the TD is found in the cisterna chyli, an abdominal structure that drains the lymphatic vessels of the intestine, pelvis, and lower limbs, and serves as the main conduit for fat absorbed by the digestive system [10]. The TD enters the thorax through the aortic hiatus, located in the posterior part of the diaphragm, and runs to the right side of the midline between the aorta and the azygos vein. It ascends through the posterior mediastinum and, at the level of T5-T6, crosses to the left of the midline, continuing superiorly before arching and ending at the junction between the left subclavian and jugular veins [10,11]. The TD can present several anatomical variations, considering that it can have many collateral canals and be drained into the intercostal veins or azygos veins [1,10]. 

**Figure 4:**
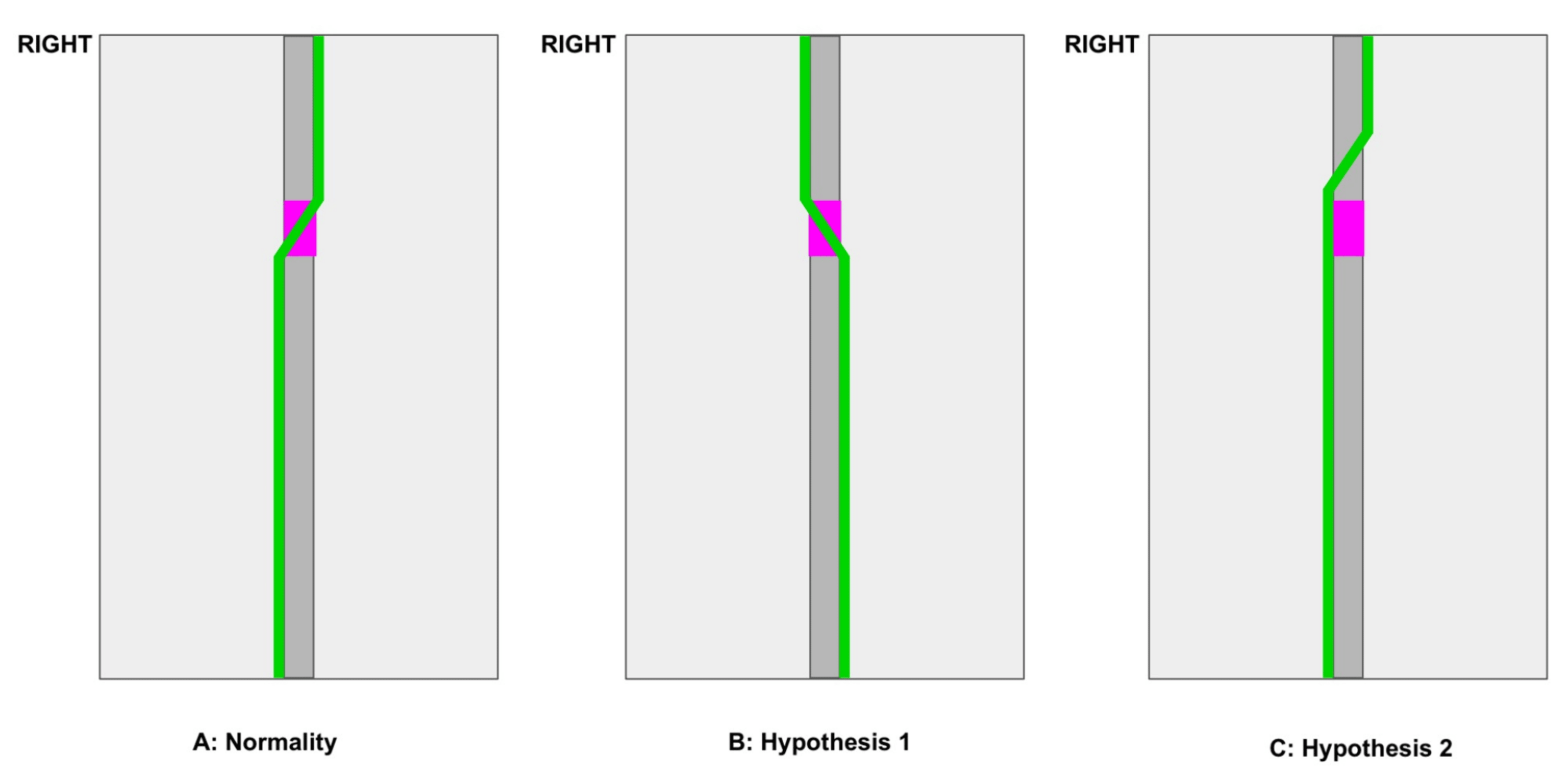
Line diagram showing the normal origin and termination of the thoracic duct, along with possible anomalous courses of the duct A: Normal anatomy: the thoracic duct (green) enters the thoracic region, in a cranial direction on the right, through the diaphragmatic hiatus and follows the spinal column, crossing to the left side between the fifth and sixth thoracic vertebrae (pink) until reaching the left subclavian artery. In the case presented, there are two possible hypotheses: B: Hypothesis 1: the thoracic duct is completely inverted, ascending paravertebrally to the left and, between the fifth and sixth vertebrae, passing to the right side and reaching the right subclavian artery. C: Hypothesis 2: the thoracic duct crosses more cranially than usual (above the fifth thoracic vertebra).

Due to these anatomical variations, the TD and its branches can be injured during thoracic surgeries, leading to the occurrence of chylothorax. Because of the anatomy of the TD's course, injuries above the thoracic plane typically cause left-sided chylothorax, while injuries below the thoracic plane usually result in right-sided chylothorax. Bilateral chylothorax can occur with injuries at the level of the thoracic plane [11]. Therefore, in the case of complications from thoracic surgery, left-sided chylothorax is commonly expected. However, in our case, due to an anomalous TD located on the right side of the chest, our patient developed right-sided chylothorax (FIGURE 4).

Preoperative imaging is essential for identifying anatomical variations of the TD, contributing significantly to the prevention of complications such as chylothorax in thoracic surgeries. This type of imaging provides essential information about the location and anatomy of the TD, helping surgeons avoid injuries during the procedure. Imaging techniques that can provide details about the anatomy of the TD include magnetic resonance ductography, computed tomography, and lymphangiography [12].

Postoperative chylothorax is suspected when chest tube drainage becomes persistent and develops a milky or turbid appearance. The diagnosis is confirmed by laboratory analysis of pleural fluid, with triglycerides measurement. A pleural fluid triglyceride concentration greater than 110 mg/dL is generally diagnostic of chylothorax [13].

Treatment options for chylothorax include conservative management with a diet enriched with medium-chain triglycerides or total parenteral nutrition, combined with effective pleural fluid drainage, somatostatin or octreotide therapy, or surgical intervention, as demonstrated in our case [14]. Dietary modification helps reduce chyle flow through the TD and promotes spontaneous closure of the leak site [13]. Drainage facilitates lung re-expansion and apposition of the pleura over the fistula, speeding up its closure. However, persistent leakage of more than 1000 mL/day, as observed in our case, indicates high-output chylothorax, with little chance of spontaneous resolution, indicating surgical treatment [15]. Definitive treatments include thoracoscopic pleurodesis, TD embolization, or TD ligation, which can be performed via open thoracotomy or video-assisted thoracoscopy [15].

Intraoperative identification of the fistula in the TD can be challenging, especially in cases with rare anatomical variations. Administering cream or oil approximately one hour before surgery, either orally or via a nasogastric tube, promotes the formation of milky fluid (chyle), which helps identify the leakage point during surgery [13]. Ligation can be performed using traditional suturing or clipping techniques. After surgical treatment, a rapid reduction in chest drain output is expected. Before removing the drain, a high-fat meal is administered to verify if the TD fistula has closed. If there is no increase in the volume of secretion, the surgical treatment is considered successful [10].

## Conclusions

In conclusion, although thoracic sympathectomy is considered a safe surgical procedure with a high success rate, uncommon complications such as chylothorax should be taken into account. Chylothorax is a rare complication after this procedure, particularly on the right side of the thorax. The TD, which can be visualized using magnetic resonance ductography or conventional lymphangiography, may exhibit anatomical variations along its course. Therefore, it is essential for thoracic surgeons to be familiar with the origin, typical course, and clinically relevant anatomical variations of the TD. In suspected cases, the anatomical variations of the TD should be considered and evaluated through imaging techniques prior to surgery, enabling early and effective diagnosis and treatment of potential chylothorax.
